# Molecular evolution of Zika virus, an neglected emerging disease in Africa and Asia

**Published:** 2011-01-10

**Authors:** Oumar Faye, Ousmane Faye, Caio César de Melo Freire, Juliana Velasco de Oliveira, Chen Rubing, Paolo M de Andrade Zanotto, Diallo Mawlouth, Amadou Alpha Sall

**Affiliations:** 1 Institut Pasteur de Dakar, Dakar, Senegal; 2 University of Sao Paulo, SP, Brazil; 3 University of Texas, Galveston, TX, USA

## 

Zika virus (ZIKV) is an arbovirus transmitted by mosquitoes isolated for the first time in Zika forest, Uganda in 1947 and repeatedly isolated in sub-Saharan Africa and South East Asia. Until 2000, only few human cases were reported but in 2007, the first major human outbreak was notified in Yap Island, Micronesia leading to 99 cases. Despite the widespread distribution of Zika virus, very limited information is available on the genetic relationship between the circulating strains. Therefore, we undertook a study on phylogeny and phylodynamics ZIKV in Africa and Asia. Partial and full length genome sequences of 38 strains from Senegal, Ivory Coast, Burkina Faso, Central African Republic and Malaysia were analysed. Phylogenetic reconstructions and datation were performed while recombination and viral population migrations were investigated. Phylogenetic analysis of the E, NS5 and NS5/3’NC gene showed two distinct ZIKV lineages circulating in Africa and a third lineage formed by the Micronesia and Malaysia strains. Besides, analysis of full length genome sequence allows identification of 5 recombinants isolates in Senegal and Ivory Coast. The 3 gene regions sequences evolved at a average rate of 7.74 x 10^-4^ nucleotide substitutions per site per year. Using the same analysis, we inferred that the most recent common ancestor of all ZIKV samples could be trace 325 years ago. The virus may have arrived in West Africa around 300 years before the present. And the migration rates showed considerable movements of ZIKV between Senegal to Ivory Coast and also to the other countries of East and central Africa. In conclusion, our study confirms previous observations showing divergences between Africa ZIKV isolate from Asia and the evidence of recombinants strains. Asian strains may represent a divergent lineage related to a common ancestor with spread throughout Southeast Asia and the Pacific from Africa.

